# Inflammatory responses to acute exercise during pulmonary rehabilitation in patients with COPD

**DOI:** 10.1007/s00421-020-04452-z

**Published:** 2020-08-07

**Authors:** Alex R. Jenkins, Neil S. Holden, Arwel W. Jones

**Affiliations:** 1 Division of Respiratory Medicine, University of Nottingham, Nottingham City Hospital, Hucknall Road, Nottingham, NG5 1PB UK; 2 School of Life Sciences, University of Lincoln, Lincoln, UK; 3 Department of Allergy, Immunology and Respiratory Medicine, Monash University, Melbourne, Australia

**Keywords:** Systemic inflammation, Exercise immunology, Lung disease

## Abstract

**Objective:**

Pulmonary rehabilitation is a cornerstone treatment in the management of chronic obstructive pulmonary disease (COPD). Acute bouts of exercise can lead to short bursts of inflammation in healthy individuals. However, it is unclear how COPD patients respond to acute bouts of exercise. This study assessed inflammatory responses to exercise in COPD patients at the start (phase 1) and end (phase 2) of pulmonary rehabilitation.

**Methods:**

Blood samples were collected before and after an acute exercise bout at the start (phase 1, *n* = 40) and end (phase 2, *n* = 27) of pulmonary rehabilitation. The primary outcome was change in fibrinogen concentrations. Secondary outcomes were changes in CRP concentrations, total/differential leukocyte counts, markers of neutrophil activation (CD11b, CD62L and CD66b), and neutrophil subsets (mature, suppressive, immature, progenitor).

**Results:**

Acute exercise (phase 1) did not induce significant changes in fibrinogen (*p* = 0.242) or CRP (*p* = 0.476). Total leukocyte count [mean difference (MD), 0.5 ± 1.1 (10^9^ L^−1^); *p* = 0.004], neutrophil count [MD, 0.4 ± 0.8 (10^9^ L^−1^); *p* < 0.001], and immature neutrophils (MD, 0.6 ± 0.8%; *p* < 0.001) increased post-exercise. Neutrophil activation markers, CD11b (*p* = 0.470), CD66b (*p* = 0.334), and CD62L (*p* = 0.352) were not significantly altered post-exercise. In comparison to the start of pulmonary rehabilitation (phase 2), acute exercise at the end of pulmonary rehabilitation led to a greater fibrinogen response (MD, 84 mg/dL (95% CI − 14, 182); *p* = 0.045).

**Conclusion:**

An acute bout of exercise does not appear to induce significant alterations in the concentrations of inflammatory mediators but can increase white blood cell subsets post-exercise. A greater fibrinogen response to acute exercise is seen at the end of pulmonary rehabilitation when compared to the start. Further research is required to understand the clinical context of these acute inflammatory responses to exercise.

## Introduction

Pulmonary rehabilitation, an exercise and education-based intervention, is considered a cornerstone treatment in the management of chronic obstructive pulmonary disease (COPD). Established benefits of pulmonary rehabilitation include inducing improvements in exercise capacity and quality of life (Nici et al. 2006). However, completion of pulmonary rehabilitation programmes is poor (Steiner et al. 2017), with a commonly cited reason for drop-out being exacerbations (Cecins et al. 2008; Fischer et al. 2009; Hayton et al. 2013; Jones et al. 2014; Keating et al. 2011). Exacerbations of COPD are acute pro-inflammatory events (Wedzicha and Donaldson 2003), and in the field of exercise immunology it has been acknowledged that acute bouts of exercise, especially if prolonged or of high intensity, can also result in short bursts of inflammation (Petersen and Pedersen 2005; Walsh et al. 2011). However, the effects of acute bouts of exercise in COPD patients are unclear whereby short bursts of inflammation resulting from exercise have been proposed to increase the risk of exacerbation (van der Vlist and Janssen 2010).

Previous research has suggested that acute exercise triggers an inflammatory response in healthy individuals as characterised by the mobilisation of neutrophils and increased concentrations of fibrinogen and CRP (Bizheh and Jaafari 2011; Montgomery et al. 1996; Nieman et al. 2005). In COPD, an acute bout of exercise has been seen to increase neutrophil counts (Van Helvoort et al. 2006). However, previous research assessing the inflammatory responses to acute exercise prior to, during and following pulmonary rehabilitation found that pulmonary rehabilitation did not affect the acute inflammatory responses of CRP (Canavan et al. 2007). Fibrinogen has recently been recognised as a reliable surrogate marker of exacerbation risk in COPD, highlighting its importance for therapeutic intervention (Duvoix et al. 2013). To date, there is no available evidence of the effects of acute exercise on fibrinogen concentrations in patients with COPD. Acute bouts of exercise have also been seen to induce changes in the expression of cell surface receptors of neutrophils in healthy populations (Gray et al. 1993; Smith et al. 1996; van Eeden et al. 1999). For example, CD62L expression, which is shed by neutrophils upon activation, has been shown to be reduced following exercise (Borregaard and Cowland 1997; Ley et al. 2007; Nathan 2006; Wittmann et al. 2004). Increases in neutrophil activation may have deleterious effects in a population such as COPD that are characterised by neutrophilic inflammation (Hoenderdos and Condliffe 2013; Quint and Wedzicha 2007).

The immunological responses to exercise in COPD may hold the key to explaining exacerbation prevention and management (Jenkins et al. 2018). Some have suggested that acute exercise may be pro-inflammatory in the early stages of an exercise training programme but continuing exercise on a regular basis may favour an anti-inflammatory response (Kasapis and Thompson 2005). In healthy populations, assessing acute responses to exercise have been suggested to have more clinical significance than training-induced alterations in resting immune/inflammatory parameters (Brandt and Pedersen 2010). However, the acute effects of exercise on inflammation in COPD patients entering a rehabilitation programme warrant further investigation.

This study aimed to assess whether acute exercise at the start of pulmonary rehabilitation in COPD patients increases markers of systemic inflammation (Phase 1). This study also aimed to assess whether acute responses to exercise differed following exercise training [e.g. end of a pulmonary rehabilitation programme (phase 2)]. The chronic basal inflammatory responses to exercise training in this cohort have been published previously (Jenkins et al. 2020). It was hypothesised that markers of systemic inflammation would increase following acute exercise during the early stages of pulmonary rehabilitation. It was also hypothesised that these effects would diminish following pulmonary rehabilitation.

## Methods

This study was conducted as part of a prospective cohort study approved by the Health Research Authority Research Ethics Committee (16/LO/0865) and registered on clinicaltrials.gov (NCT02740686). All patients provided written consent.

### COPD patients

To assess responses to acute exercise at the start (phase 1) and end (phase 2) of a conventional community pulmonary rehabilitation programme, 40 COPD patients (age 69 ± 7 years; FEV_1_ predicted 51 ± 17%) were recruited from the National Health Service in the UK. Recruited COPD patients were clear of an exacerbation for 4 weeks prior to sampling (i.e. not being treated with antibiotics or steroids). COPD patients presenting with a clinical diagnosis of concurrent active inflammatory (e.g. rheumatoid arthritis or cancer) or respiratory conditions (e.g. pulmonary fibrosis, asthma or bronchiectasis) were excluded from this study.

### Procedures

COPD patients performed exercises as part of a conventional community pulmonary rehabilitation programme involving two supervised exercise sessions a week for 8-weeks utilising minimal resources. Each session consisted of 1 h of exercise and 1 h of education. Exercise targets were adjusted for each session by the physiotherapist depending on performance, progression and health status of each patient with the aim of progressing workloads. Exercises were interchangeable targeting cardiorespiratory fitness (e.g. shuttle walking, step-ups, get-up-and-go) and muscular strength (e.g. bicep curls, wall press, bent arm lateral raise, and cross and reach) with targets set for achieving 4 to 5 on the Borg Dyspnoea Scale after each exercise. No dietary interventions or restrictions were put in place for this study.

**Fig. 1:**
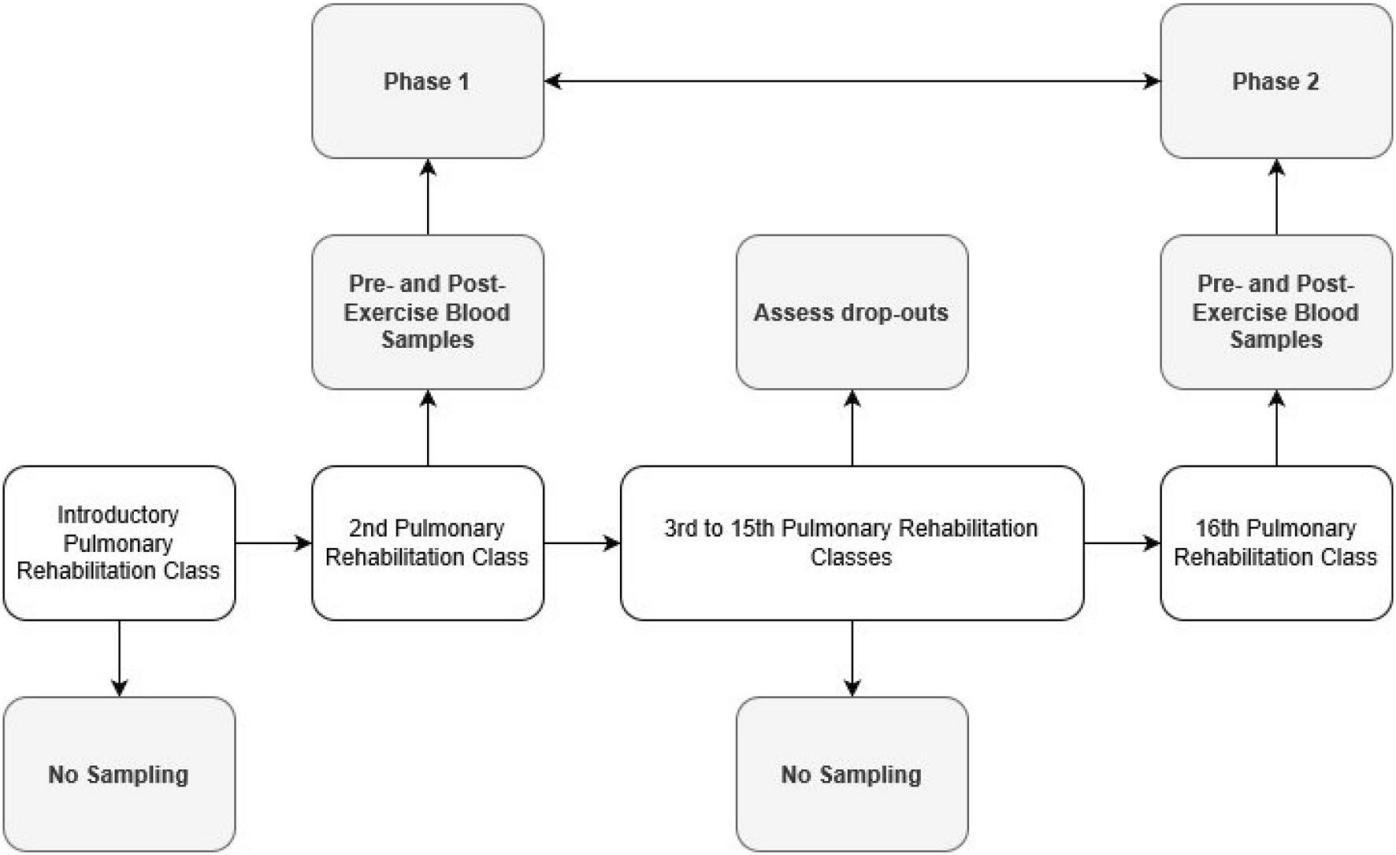
Study procedures

Blood samples were taken before and after an acute bout of exercise at the beginning (phase 1, 2nd session of programme) and end (phase 2, 16th session) of the pulmonary rehabilitation programme, providing patients did not dropout of the course. The 2nd class of the programme was chosen for assessment of acute responses to exercise, as the 1st class of pulmonary rehabilitation is an induction/familiarisation session where the performing of exercises is limited (Fig. 1).

The primary outcome of fibrinogen concentration (sodium citrate) and secondary outcome of CRP concentration (K_3_EDTA) were quantified in plasma through the use of ELISAs (AssayPro LLC, Missouri, USA). Secondary outcomes of total/differential leukocyte counts were quantified via haematological analysis (ABX Pentra 60C+ , HORIBA Medical, Montpellier, France). Further secondary measures were undertaken on a subset of patients (*n* = 20) whole blood to assess markers of neutrophil activation (CD11b, CD62L, CD66b) and neutrophil phenotypes (mature, CD16^high^/CD62L^high^; suppressive, CD16^high^/CD62L^low^; immature, CD16^low^/CD62L^high^; progenitor, CD16^low^/CD62L^low^) with the use of flow cytometry (FACSVerse, Becton Dickinson, NJ, USA).

### Statistical analysis

Phase 1 data were analysed as absolute values (pre- vs. post-exercise) whereas Phase 2 data were analysed as mean differences between pre- and post-exercise (beginning vs. end of pulmonary rehabilitation). In phase 1 (pre- vs. post-exercise), changes in fibrinogen and CRP concentrations, total/differential cell counts, neutrophil activation markers (CD11b, CD62L, CD66b), and neutrophil maturity markers (mature, CD16^high^/CD62L^high^; suppressive, CD16^high^/CD62L^low^; immature, CD16^low^/CD62L^low^; progenitor, CD16^low^/CD62L^low^) following acute exercise were analysed using one-tailed paired t-tests. In phase 2 (beginning vs. end of rehabilitation), differences in fibrinogen and CRP concentrations, total/differential cell counts, neutrophil activation markers (CD11b, CD62L, CD66b), and neutrophil maturity markers (mature, CD16^high^/CD62L^high^; suppressive, CD16^high^/CD62L^low^; immature, CD16^low^/CD62L^high^; progenitor, CD16^low^/CD62L^low^) responses to acute exercise across bouts were analysed using one-tailed paired t-tests. Statistical significance was accepted at *p* < 0.05.

## Results

### Phase 1

#### Patient characteristics

The characteristics of the recruited COPD patients are detailed in Table 1. Most COPD patients were categorised as GOLD grade D (65%) with a lesser proportion classified as GOLD grade B (33%). The majority of COPD patients were categorised as either mMRC grade 2, grade 3, or grade 4. The average duration of the acute exercise bout was 26 ± 4 min.

**Table 1:** Patient characteristics (phases 1 and 2)

Variable	COPD, phase 1 (*n* = 40)	COPD, phase 2 (*n* = 27)
Age (years)^a^	69 ± 7	69 ± 7
% Males^b^	58%	56%
Body mass (kg)^a^	77 ± 17	74 ± 15
GOLD grade, *n* (%)^c^		
A	1 (2)	1 (4)
B	13 (33)	10 (37)
C	0 (0)	0 (0)
D	26 (65)	16 (59)
FEV_1_% predicted^a^	51 ± 17	50 ± 16
Charlson Comorbidity Index^a^	3.9 ± 1.1	4.0 ± 1.2
Current smokers^b^	23%	19%
Oxygen users^b^	15%	11%
Hospitalisations (past 12 months)^a^	0.5 ± 0.7	0.4 ± 0.7
Exacerbations (past 12 months)^a^	2.2 ± 1.6	2.1 ± 1.6
Daily beclomethasone equivalent (µg)^a^	860 ± 540	889 ± 621
mMRC, *n* (%)^c^		
0	1 (2)	1 (4)
1	0 (0)	0 (0)
2	6 (15)	4 (15)
3	12 (30)	9 (33)
4	21 (53)	13 (48)
Average exercise duration (min)^a^	26 ± 4	40 ± 4

^a^Data presented as mean ± SD

^b^Data presented as a % of total population

^c^Data presented as total number

### Phase 1

#### Inflammatory responses

**Fig. 2:**
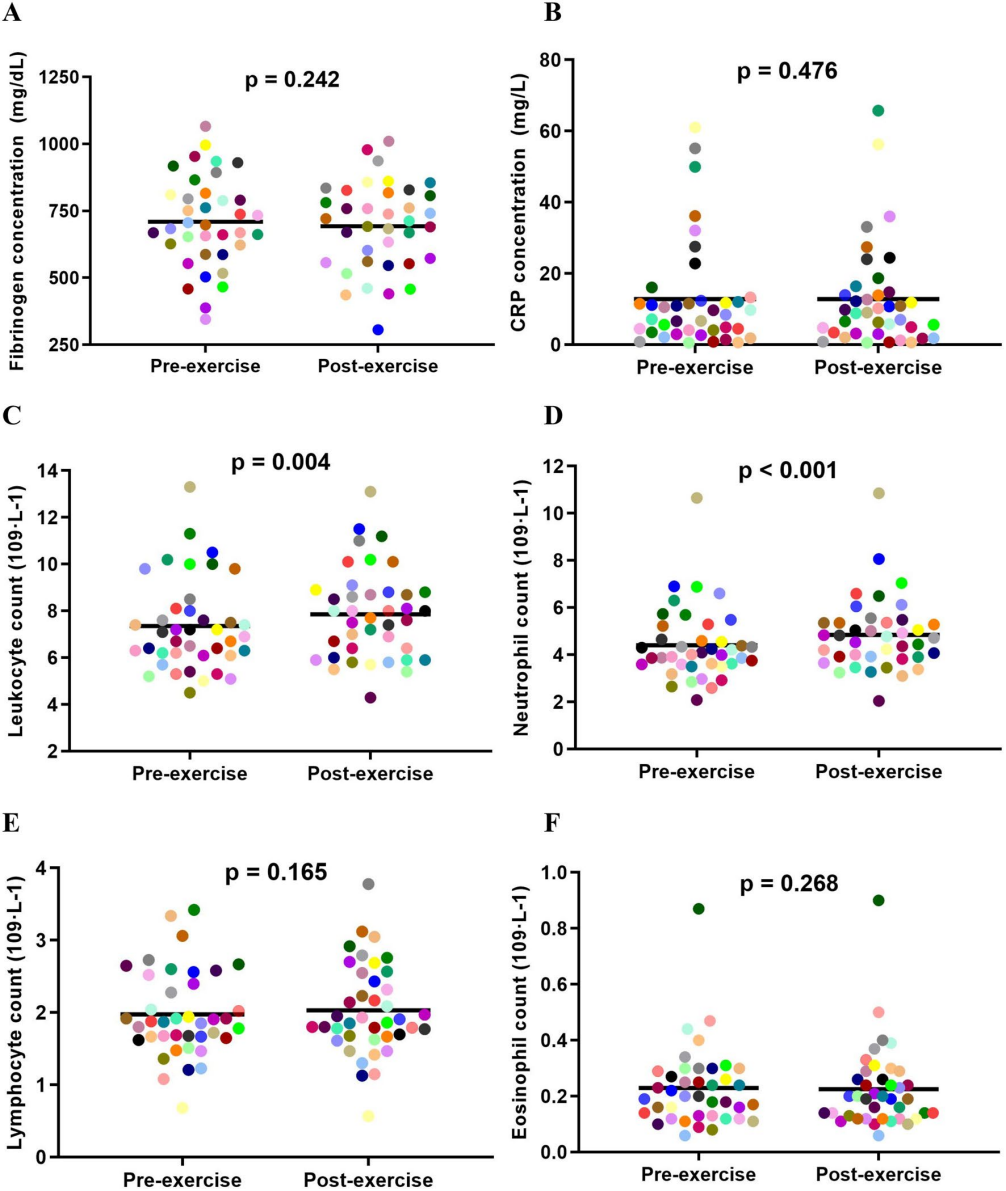
Fibrinogen (**a**), CRP (**b**), total leukocyte (**c**), neutrophil (**d**), lymphocyte (**e**), and eosinophil (**f**) responses to an acute bout of exercise at the beginning of pulmonary rehabilitation (phase 1)

There were no significant changes in fibrinogen (*p* = 0.242), CRP (*p* = 0.476), lymphocytes (*p* = 0.165), or eosinophils (*p* = 0.268) following an acute bout of exercise at the beginning of pulmonary rehabilitation. However, there were significant increases in total leukocyte (*p* = 0.004) and blood neutrophil (*p* < 0.001) counts following an acute bout of exercise. (Fig. 2).

#### Neutrophil surface expression

There were no significant changes in blood neutrophil surface CD11b (*p* = 0.470), CD62L (*p* = 0.352), or CD66b (*p* = 0.334) expression following an acute bout of exercise at the beginning of pulmonary rehabilitation (Table 2).

**Table 2:** Responses of neutrophil activation markers and maturity phenotypes to acute exercise at the beginning of pulmonary rehabilitation

Neutrophil marker	2nd pre (*n* = 20)	2nd post (*n* = 20)	*p* values
CD11b (MFI)	5508 ± 791	5495 ± 748	0.470
CD62L (MFI)	26,852 ± 6185	26,534 ± 5523	0.352
CD66b (MFI)	9447 ± 2956	9604 ± 2930	0.334
CD16b^high^/CD62L^high^ (%)	92.6 ± 3.0	92.6 ± 2.2	0.414
CD16b^high^/CD62L^low^ (%)	5.5 ± 3.2	4.9 ± 2.5	0.073
CD16b^low^/CD62L^low^ (%)	0.1 ± 0.1	0.2 ± 0.2	0.210
CD16b^low^/CD62L^high^ (%)	1.7 ± 0.8	2.3 ± 1.1	< 0.001

Data presented as mean ± SD. *MFI* median fluorescent intensity. Thirteen COPD patients were excluded from these analyses due to a lack of expression of CD16b meaning that neutrophils could not be confidently isolated for analysis. A further seven COPD patients did not provide blood samples for analysis of neutrophil activation markers

The mature (*p* = 0.414), suppressive (*p* = 0.073), and progenitor (*p* = 0.210) neutrophil phenotypes were not significantly changed as a result of an acute bout of exercise at the beginning of pulmonary rehabilitation. However, the immature neutrophil subset (*p* < 0.001) was significantly increased following an acute bout of exercise at the beginning of pulmonary rehabilitation (Table 2).

### Phase 2

#### Patient characteristics

From phase 1, there were ten COPD patients who did not go on to complete pulmonary rehabilitation with a further three patients lost due to treatment for exacerbations at the end of pulmonary rehabilitation.

The characteristics of the COPD patients who completed phase 2 are detailed Table 1. A large proportion of patients were categorised as GOLD grade D (59%) with a lesser proportion classified as GOLD grade B (37%). The majority of COPD patients were categorised as either mMRC grade 2, grade 3, or grade 4. The duration of the acute exercise bout at the end of pulmonary rehabilitation was 40 ± 4 min.

#### Inflammatory responses

Although, there was not a significant change in fibrinogen in phase 1, there was a significant difference in changes between the conditions (i.e. pre-rehab exercise bout vs. post-rehab exercise bout) (*p* = 0.045) when comparing fibrinogen responses to acute exercise.

No significant effects of condition were observed when comparing CRP (*p* = 0.483), leukocyte count (*p* = 0.159), neutrophil count (*p* = 0.279), eosinophil count (*p* = 0.155), and lymphocyte count (*p* = 0.119) responses to acute exercise between bouts taking place at the start and end of pulmonary rehabilitation (Table 3).

**Table 3:** Comparisons of mean differences in systemic inflammatory responses to acute bouts of exercise at the beginning and end of pulmonary rehabilitation

Variable	2nd class		16th class		Difference in classes for change post-exercise	*p* values
	Pre	Post	Pre	Post		
Fibrinogen (mg/dL), *n* = 25^a^	694 ± 189	654 ± 181	588 ± 197	632 ± 191	84 (− 14, 182)	0.045*
CRP (mg/L), *n* = 27	13.3 ± 15.0	14.2 ± 16.0	14.2 ± 14.4	15.0 ± 16.5	− 0.1 (− 2.8, 2.7)	0.483
Total Leukocytes (10^9^ L^−1^), *n* = 27	7.12 ± 1.81	7.48 ± 1.60	6.70 ± 1.24	7.39 ± 1.51	0.33 (− 0.33, 0.98)	0.159
Neutrophils (10^9^ L^−1^), *n* = 27	4.22 ± 1.24	4.61 ± 1.13	3.90 ± 1.02	4.43 ± 1.19	0.14 (− 0.33, 0.60)	0.279
Eosinophils (10^9^ L^−1^), *n* = 27	0.21 ± 0.11	0.20 ± 0.10	0.20 ± 0.11	0.20 ± 0.11	− 0.01 (− 0.03, 0.01)	0.155
Lymphocytes (10^9^ L^−1^), *n* = 27	1.93 ± 0.61	1.91 ± 0.56	1.90 ± 0.54	1.97 ± 0.54	0.09 (− 0.06, 0.24)	0.119

Data expressed as mean ± SD or mean difference (95% CI)

^a^Plasma samples unable to be obtained from two COPD patients for measurement of fibrinogen

*Significant difference between bouts in fibrinogen response to exercise (*p* < 0.05)

### Neutrophil surface expression

There were no significant differences between conditions in neutrophil surface CD11b (*p* = 0.236), CD62L (*p* = 0.195), or CD66b (*p* = 0.481) responses to acute bouts of exercise taking place at the beginning and end of pulmonary rehabilitation (Table 4).

**Table 4:** Comparison of mean differences for neutrophil activation markers and maturity phenotypes in response to acute exercise at the beginning and end of pulmonary rehabilitation

Neutrophil marker	2nd class		16th class		Difference in classes for change post-exercise	*p* values
	Pre	Post	Pre	Post		
CD11b (MFI)	5390 ± 756	5469 ± 822	5744 ± 855	5673 ± 845	− 150 (− 584, 284)	0.236
CD62L (MFI)	25,627 ± 5320	25,711 ± 5285	28,293 ± 5427	27,341 ± 6770	− 1036 (− 3526, 1453)	0.195
CD66b (MFI)	9179 ± 3079	9334 ± 3033	10,364 ± 1496	10,501 ± 1577	− 18 (− 815, 780)	0.481
CD16b^high^/CD62L^high^ (%)	92.49 ± 3.41	92.34 ± 2.37	92.45 ± 2.34	91.51 ± 1.91	− 0.79 (− 1.98, 0.39)	0.086
CD16b^high^/CD62L^low^ (%)	5.80 ± 3.55	5.34 ± 2.61	5.43 ± 2.42	5.84 ± 2.05	0.87 (− 0.21, 1.94)	0.053
CD16b^low^/CD62L^low^ (%)	0.14 ± 0.13	0.15 ± 0.09	0.17 ± 0.23	0.16 ± 0.09	− 0.02 (− 0.18, 0.16)	0.454
CD16b^low^/CD62L^high^ (%)	1.57 ± 0.69	2.16 ± 1.09	1.95 ± 1.41	2.49 ± 1.70	− 0.05 (− 0.33, 0.24)	0.363

Data expressed as mean ± SD or mean difference (95% CI). *MFI* median fluorescent intensity. Of the 20 COPD patients measured in phase 1, four were lost in the follow-up for the measurement of neutrophil activation markers

The mature (*p* = 0.086), immature (*p* = 0.363), suppressive (*p* = 0.053), and progenitor (*p* = 0.454) neutrophil phenotypes were not significantly different between conditions in terms of acute responses to exercise taking place at the beginning and end of pulmonary rehabilitation (Table 4).

## Discussion

The purpose of this study was to assess inflammatory responses to acute bouts of exercise at the beginning (phase 1) and end (phase 2) of pulmonary rehabilitation to see how inflammatory responses to exercise differ with exercise training progression in patients with COPD. The main findings from this study suggest that acute exercise in COPD patients at the start of pulmonary rehabilitation does not result in significant increases in circulating concentrations of fibrinogen and CRP. However, increases in total leukocyte and neutrophil counts, which was accompanied by an increase in the proportion of immature neutrophils, were observed following this initial acute bout of exercise. Differences between fibrinogen responses to acute exercise at the start and end of a pulmonary rehabilitation programme were statistically significant. No statistically significant differences in CRP, total leukocyte, and neutrophil count responses to acute exercise were observed between the start and the end of rehabilitation.

This is the first study in the COPD population to assess fibrinogen concentrations in response to acute exercise bouts at the beginning and end of pulmonary rehabilitation. The present findings suggest that an acute bout of exercise at the beginning of a traditional pulmonary rehabilitation programme is not sufficient to significantly alter the concentrations of fibrinogen. These findings disagree with previous research in other clinical populations (e.g. atrial fibrillation) whereby fibrinogen levels were observed to increase following an acute bout of exercise (Li-Saw-Hee et al. 2001). It is important to note this previous study utilised strenuous exercise to assess fibrinogen responses (Li-Saw-Hee et al. 2001). In the current study, however, between conditions (pre-rehabilitation vs. post-rehabilitation), there was a significant difference whereby a greater fibrinogen response to acute exercise was seen at the end of rehabilitation. This potentially reflects the progressive nature of exercise training within pulmonary rehabilitation whereby the absolute intensity and/or duration of exercise at the beginning of the course may not be sufficient to induce an inflammatory response of fibrinogen. Therefore, it may be deemed that exercise needs to be strenuous and/or prolonged in nature to induce increases in fibrinogen concentration. These areas warrant further research to identify at what stage in a pulmonary rehabilitation programme there is a change in response of fibrinogen to acute exercise. Nevertheless, the findings indicate that acute exercise at the beginning of pulmonary rehabilitation does not exacerbate a key marker of systemic inflammation in COPD patients.

This study also found that CRP was not significantly elevated following acute bouts of exercise at the start and end of pulmonary rehabilitation. This agrees with previous research in the context of pulmonary rehabilitation whereby CRP was not seen to be elevated following acute bouts of exercise at the beginning and end of pulmonary rehabilitation (Canavan et al. 2007). The conclusions from the study suggested that pulmonary rehabilitation was unlikely to enhance systemic inflammation (Canavan et al. 2007).

The findings of the current study demonstrate changes in immune cell trafficking, namely increases in total leukocyte counts and neutrophil counts, regardless of whether an exercise bout is completed at the beginning or end of pulmonary rehabilitation. This agrees with well-established literature assessing acute immunological responses to exercise in the healthy population (Brown et al. 2015; Nieman et al. 1991; Quindry et al. 2003) and the limited existing evidence in COPD populations (Menon et al. 2012; van Helvoort et al. 2005). While it is not surprising to see these effects, it could be hypothesised that the acute inflammatory response to exercise could diminish by the end of pulmonary rehabilitation as a result of exercise training (Beavers et al. 2010). This was not observed in the current study, which may suggest that the relative exercise intensity was maintained throughout the programme. However, it would be insightful to assess inflammatory responses to two controlled bouts of exercise, in terms of intensity and duration, before and after an exercise training programme as recently undertaken (Silva et al. 2018).

There were some interesting observations for neutrophil activation markers in the context of acute exercise in COPD patients in the current study. The findings disagree with that of previous literature whereby reductions in CD62L expression (Kurokawa et al. 1995; van Eeden et al. 1999) as well as increases in CD11b and CD66b (Pizza et al. 1996; Smith et al. 1996; van Eeden et al. 1999) were not observed post-exercise in COPD patients. The data presented in the current study also show that these neutrophil activation marker responses to acute exercise are not susceptible to significant change following a period of exercise training.

This study was also the first study to utilise the newly proposed phenotyping approach of neutrophils in an exercise setting with a COPD population (Cortjens et al. 2017). An acute bout of exercise appeared to increase the percentage of immature neutrophils following exercise at the beginning of pulmonary rehabilitation with similar responses observed following a period of pulmonary rehabilitation. To our knowledge, this approach has not yet been adopted in the field of exercise immunology so comparative literature is not available. However, the findings support the notion long proposed in healthy populations that the physical stress of exercise induces an inflammatory response and a temporary neutrophilia as a result of increased release of immature neutrophils from the bone marrow (Suzuki et al. 1999; Yamada et al. 2002). However, these responses did not translate to any significant changes within any of the other neutrophil subsets. It would be insightful to utilise this phenotypic approach in future to further explore the effects of acute exercise on neutrophil subset responses.

When interpreting the findings of the current study, it is important to consider some limitations. One aspect not accounted for in this study was the baseline physical activity status of the participants. Physically active lifestyles have been seen to alleviate some of the inflammatory responses to an acute bout of exercise (Gokhale et al. 2007; Woods et al. 2012). However, the effects of this in the current study are potentially minimal as the majority of COPD patients are inactive upon referral to pulmonary rehabilitation. Another important factor to consider is that the exercise workloads were not matched between bouts at the beginning and end of pulmonary rehabilitation. The duration (26 min vs. 40 min) was not the same between the two timepoints, but the modality of exercise and intended relative intensity were kept consistent. In a real world clinical pulmonary rehabilitation setting such as pulmonary rehabilitation including the presentation of a diverse range of COPD patients, it is hard to control the intensities and rates of progression of exercises for the patients in each group. However, the data presented are still purposeful in detecting changes to treatment in a real-world setting. It is also important to highlight that acute inflammatory responses to exercise were assessed immediately post-exercise, and there is a need for further research to assess any delayed inflammatory responses to exercise (e.g. 1 h–24 h post-exercise). Interpretations of the results also need to take into account the drop-out between phases 1 and 2, which is commonly observed with an intervention such as pulmonary rehabilitation (Steiner et al. 2018), whereby missing data introduce an element of bias with the results. However, it is worth noting that the patient cohorts in both phases appeared clinically homogenous in terms of the measured characteristics. Finally, the sample size used to make inferences about the neutrophil activation markers was impacted by an unforeseen deficiency of CD16b expression in subsets of patients. This reduced the confidence to be able to separate neutrophils from eosinophils; hence, participants were removed from the analysis. Research has previously suggested two possible causes for this phenomena; a rare hereditary deficiency of CD16 (Wagner and Hansch 2004) or a shedding of CD16b via the action of ADAM17 (Wang et al. 2013). These proposals could not be assessed in the current study and future studies should be wary of the use of CD16b as a marker for identifying neutrophils in COPD.

This study implies that acute exercise does not appear to worsen selected key systemic inflammatory markers in COPD patients as characterised by a lack of increase in established biomarkers of exacerbations in the initial stages of pulmonary rehabilitation (fibrinogen and CRP). Acute exercise does appear to induce classical changes in leukocyte trafficking, characterised by increases in total leukocyte and neutrophil counts as observed previously in healthy (Brown et al. 2015; Nieman et al. 1991; Quindry et al. 2003) and COPD populations (Menon et al. 2012; van Helvoort et al. 2005). Importantly, such leukocyte trafficking occurs independent of the time at which the acute exercise bout was undertaken in a pulmonary rehabilitation programme. These findings warrant further follow-up in COPD patients via assessment of acute inflammatory responses in exercise bouts controlled for both duration and intensity.

## Abbreviations

COPD: Chronic obstructive pulmonary disease
CRP: C-reactive protein
ELISA: Enzyme-linked immunosorbent assay
FEV_1_: Forced expiratory volume in 1 s
GOLD: Global initiative for chronic obstructive lung disease
mMRC: Modified Medical Research Council
UK: United Kingdom
